# Clearance of the mutant androgen receptor in motoneuronal models of spinal and bulbar muscular atrophy^☆^

**DOI:** 10.1016/j.neurobiolaging.2013.05.026

**Published:** 2013-11

**Authors:** Paola Rusmini, Valeria Crippa, Elisa Giorgetti, Alessandra Boncoraglio, Riccardo Cristofani, Serena Carra, Angelo Poletti

**Affiliations:** a Sezione di Biomedicina e Endocrinologia, Dipartimento di Scienze Farmacologiche e Biomolecolari (DiSFeB), Centro di Eccellenza sulle Malattie Neurodegenerative, Universita' degli Studi di Milano, Milan, Italy; b Centro InterUniversitario sulle Malattie Neurodegenerative, Universita' degli Studi di Firenze, Milano, Genova e Roma Tor Vergata, Sede di Milano, Italy; c Sezione di Fisiologia e Neuroscienze, Dipartimento di Scienze Biomediche, Metaboliche e Neuroscienze, Università degli Studi di Modena e Reggio Emilia, Modena, Italy

**Keywords:** Spinal and bulbar muscular atrophy, Androgen receptor, Polyglutamine, CAG repeat, Motoneuron disease, Motoneuron, Neurodegeneration, Protein misfolding, Chaperones, HspB8

## Abstract

Spinal and bulbar muscular atrophy (SBMA) is an X-linked motoneuron disease caused by an abnormal expansion of a tandem CAG repeat in exon 1 of the androgen receptor (AR) gene that results in an abnormally long polyglutamine tract (polyQ) in the AR protein. As a result, the mutant AR (ARpolyQ) misfolds, forming cytoplasmic and nuclear aggregates in the affected neurons. Neurotoxicity only appears to be associated with the formation of nuclear aggregates. Thus, improved ARpolyQ cytoplasmic clearance, which indirectly decreases ARpolyQ nuclear accumulation, has beneficial effects on affected motoneurons. In addition, increased ARpolyQ clearance contributes to maintenance of motoneuron proteostasis and viability, preventing the blockage of the proteasome and autophagy pathways that might play a role in the neuropathy in SBMA. The expression of heat shock protein B8 (HspB8), a member of the small heat shock protein family, is highly induced in surviving motoneurons of patients affected by motoneuron diseases, where it seems to participate in the stress response aimed at cell protection. We report here that HspB8 facilitates the autophagic removal of misfolded aggregating species of ARpolyQ. In addition, though HspB8 does not influence p62 and LC3 (two key autophagic molecules) expression, it does prevent p62 bodies formation, and restores the normal autophagic flux in these cells. Interestingly, trehalose, a well-known autophagy stimulator, induces HspB8 expression, suggesting that HspB8 might act as one of the molecular mediators of the proautophagic activity of trehalose. Collectively, these data support the hypothesis that treatments aimed at restoring a normal autophagic flux that result in the more efficient clearance of mutant ARpolyQ might produce beneficial effects in SBMA patients.

## 1 Introduction

Spinal and bulbar muscular atrophy (SBMA) is an X-linked neuromuscular disease characterized by the loss of motoneurons in the spinal cord and in the bulbar regions of the brain stem. Degeneration of sensory neurons in the dorsal root ganglia has also been reported. SBMA is the result of a CAG triplet repeat expansion in the androgen receptor (AR) gene (La Spada et al., 1991) that results in an elongated polyglutamine (polyQ) tract in the N-terminal transactivation domain of the mutant AR protein (ARpolyQ). In unaffected individuals, the polyQ tract ranges in size from 9 to 37 Qs, and polyQ expansions that extend longer than 38 contiguous Qs lead to neuronal toxicity that is the cause of motoneuron death in SBMA. Eight other inherited neurodegenerative diseases have been found to be the result of similar elongated polyQ tracts and are classified as CAG/polyQ-diseases (Orr and Zoghbi, 2007; Pennuto et al., 2009). In each disease, the elongated polyQ tract is believed to act through a gain-of-function mechanism by inducing the formation of an aberrant protein conformation. This leads to protein misfolding and aggregation, and ultimately neuronal toxicity (motoneuronal in the case of SBMA). The fact that AR is an extremely well characterized member of the steroid receptor superfamily (Poletti, 2004; Vismara et al., 2009) makes SBMA a valuable model to study CAG/polyQ-related diseases because it allows for the discrimination between the physiological and pathological activities of ARpolyQ. In addition, ARpolyQ toxicity strictly depends on the binding of the receptor to androgens (Chevalier-Larsen et al., 2004; Fischbeck, 2012; Katsuno et al., 2003a, 2012; Schmidt et al., 2002), which apparently converts ARpolyQ from a non-pathological to a pathological status (Katsuno et al., 2003a, 2003b), possibly via the induction of conformational changes in the mutant protein. These alterations could be linked to the release of accessory chaperones and/or to the AR nuclear translocation, because both processes have been linked to ARpolyQ neurotoxicity. Incorrect ARpolyQ folding during its activation results in the formation of nuclear aggregates in anterior horn spinal cord motoneurons and cytoplasmic aggregates in dorsal root ganglia (Adachi et al., 2005; Suzuki et al., 2008). Nuclear localization, but not aggregation, is apparently required for ARpolyQ neurotoxicity (Montie et al., 2009, 2011). In fact, cytoplasmic retention of ARpolyQ correlates with a decrease in its toxicity (Montie et al., 2009, 2011). However, non-nuclear ARpolyQ neurotoxic mechanisms have also been described (Morfini et al., 2006; Piccioni et al., 2002; Ranganathan et al., 2009; Schindler et al., 2012); for instance, misfolded cytoplasmic ARpolyQ can trigger a Bax-dependent apoptotic pathway (Young et al., 2009). In our motoneuronal SBMA models, testosterone induces the formation of cytoplasmic ARpolyQ aggregates (Simeoni et al., 2000) that we have already shown do not correlate with increased cell death (Rusmini et al., 2007; Simeoni et al., 2000). We postulated that, at least in the early stages of their formation, cytoplasmic ARpolyQ aggregation contributes to segregate the harmful proteins into physically defined intracellular compartments (Carra et al., 2012). At later stages, however, these aggregates might themselves become toxic because they might affect other cellular activities (e.g., protein clearance, axonal transport) that might also contribute to neurotoxicity (Piccioni et al., 2002; Poletti, 2004; Rusmini et al., 2010).

Apart from their role in neurotoxicity, aggregates represent a valuable “marker of misfolding” because their presence clearly correlates with defects in the misfolded protein clearance via proteolytic pathways (both the ubiquitin-proteasome system [UPS] or autophagy) (Carra et al., 2012; Rusmini et al., 2010, 2011; Sau et al., 2011). Indeed, cytoplasmic soluble ARpolyQ (testosterone-untreated) has been shown to impair UPS activity (Rusmini et al., 2007) because it might overwhelm its degradative capacity. In addition, it has been shown that the UPS poorly degrades long polyQs (Holmberg et al., 2004). Interestingly, ARpolyQ aggregation (induced by testosterone) correlates with UPS desaturation (Rusmini et al., 2007). In addition, ARpolyQ (with or without testosterone) activates autophagy (Rusmini et al., 2010) and autophagic inhibition increases ARpolyQ aggregation (Rusmini et al., 2010). Thus, cytoplasmic ARpolyQ aggregation might depend on autophagic flux failure (Rusmini et al., 2010) so that the stimulation of autophagy would have beneficial effects.

We recently showed that the small heat shock protein B8 (HspB8) facilitates autophagic removal of other proteins associated with motoneuron diseases (e.g., SOD1 and TDP-43) (Carra et al., 2012; Crippa et al., 2010a, 2010b). Moreover, it has been already shown that HspB8 exerts antiaggregation and/or prodegradative activity on ARpolyQ, but so far this has been evaluated only on the AR nontoxic conformational status (without testosterone) (Carra et al., 2005). Here, we evaluated and compared the HspB8 activities on ARpolyQ clearance in the presence and absence of testosterone and found that HspB8 restores the normal autophagic flux in motoneuronal cells expressing ARpolyQ (with or without testosterone). Interestingly, we also found that the trehalose proautophagic activity might be mediated by HspB8 action.

## 2 Methods

Chemicals, 3-methyladenine (3-MA), MG132, and trehalose were obtained from Sigma-Aldrich (St Louis, MO, USA).

### 2.1 Plasmids

The plasmids AR.Q23 and AR.Q46, routinely used in our laboratory, have been previously described (Simeoni et al., 2000). The GFP-AR.Q22 and GFP-AR.Q48 were obtained by insertion of AR cDNA into the green fluorescent protein (GFP) vector, expressing chimeric fluorescent fusion proteins as previously described (Stenoien et al., 1999). The plasmid pCNEO-cMyc-HspB8 expressing the *myc* tagged-rat HspB8 was obtained from J. Landry, Université Laval, Québec, Canada (Chavez Zobel et al., 2003) and the plasmid pCI-HspB8 contains the sequence of human HspB8 cloned in pCI (Promega, Madison, WI, USA) (Carra et al., 2005). The plasmid GFPu, expressing a proteasome activity reporter based on a GFP fused to a constitutive degradation signal (CL-1), was obtained from Ron Kopito, Stanford University, Stanford, CA, USA (Bence et al., 2001). The plasmid pmRFP-LC3, expressing the autophagic reporter LC3 tagged with mRFP, was obtained from Aviva Tolkovsky, University of Cambridge, UK (Klionsky et al., 2012). The plasmid pDest-mCherry-p62, expressing a Cherry-tagged p62, was obtained from Terje Johansen, University of Tromsø, Norway (Pankiv et al., 2007). pDsRed-monomer-C1 encodes a monomeric mutant form of the *Discosoma sp*. Red fluorescent protein DsRed (Clontech Laboratories, Inc, Mountain View, CA, USA). The plasmid promB8 expresses the firefly luciferase gene under the control of the human HspB8 promoter (Crippa et al., 2010b). pRL-TK was from Promega. To silence LC3 expression we used an shRNA construct against *Mus Musculus* Map1LC3b in pGFP-V-RS-vector and a scrambled noneffective shRNA, as control (OriGene Technologies, Inc, Rockville, MD, USA), as previously described (Rusmini et al., 2011).

To silence endogenous HspB8 expression we used a custom siRNA duplex (CGG AAG AGC UGA UGG UAA AUU) (Dharmacon, Thermo Scientific Life Sciences Research, Waltham, MA, USA).

### 2.2 Cell cultures and transfections

The immortalized motoneuron cell line, NSC34 (Cashman et al., 1992), is routinely used in our laboratory (Crippa et al., 2010a, 2010b; Piccioni et al., 2001; Rusmini et al., 2011; Simeoni et al., 2000), and was transfected with lipofectamine (Invitrogen, Life Technologies Corporation, Carlsbad, CA, USA) and transferrin (Sigma-Aldrich), as previously described (Marron et al., 2005; Simeoni et al., 2000) using 1.0–1.5 μg of plasmid DNA, 3 μL of transferrin solution and 2 μL of lipofectamine.

HspB8 and scramble siRNA were transfected using Lipofectamine 2000 (Invitrogen) according to the manufacturers' instructions.

In the experiments involving steroid hormone treatments, the fetal bovine serum (FBS) was replaced with charcoal stripped-FBS, to eliminate endogenous steroids (Poletti et al., 2001; Pozzi et al., 2003).

The following amounts of cDNAs were used: (1) 0.5 μg of AR.Q(n) or GFP-AR.Q(n); (2) 0.6 μg of pCNEO-cMyc-HspB8, pCIHspB8 or pCDNA3 as vector control; (3) 0.05 μg of GFPu; (4) 0.2 μg of DsRed Monomer; (5) 0.2 μg of mRFP-LC3; (6) 0.2 μg of mCherry-p62; (7) 0.5 μg of shRNA against LC3 or shRNA scrambled control; (8) 0.25 μg of promB8; and (9) 0.25 μg of pRL-TK.

### 2.3 Fluorescence, immunofluorescence, and microscopy on NSC34 cells

NSC34 cells were plated in 12-well multiwell plates containing coverslips at a density of 70,000 cells per well and transiently transfected as previously described. The cells were allowed to grow for 48 hours and then fixed and processed as previously described (Sau et al., 2007). To analyze AR.Q(n), HspB8, LC3, and p62, we used the following primary antibodies: rabbit polyclonal AR (H280) (Santa Cruz Biotech, SantaCruz, CA, USA) 1:100 in milk, rabbit polyclonal anti-HspB8 antibody 1:400 in 3% BSA Tween-TBS (TBS-T, 20 mM TrisHCl, pH 7.5, 0.5 M NaCl, 0.05% Tween-20) (Carra et al., 2005), rabbit polyclonal anti-LC3-B antibody 1:1000 (Sigma-Aldrich) in milk, rabbit polyclonal anti-SQSTM1/p62 antibody 1:1000 (Abcam, Cambridge, UK) in milk. Secondary antibodies used were: Alexa 488 anti-rabbit and Alexa 594 anti-rabbit (Invitrogen) 1:1000 in milk. Cells were stained with DAPI to visualize the nuclei. An Axiovert 200 microscope (Zeiss, Oberkochen, Germany) equipped with FITC/TRITC/DAPI and combined with a Photometric Cool-Snap CCD camera (Ropper Scientific, Trenton, NJ, USA) was used. Images were processed using Metamorph software, version 7.7.7.0 (Universal Imaging, Downingtown, PA, USA).

GFP-AR.Q48 transfected cells bearing aggregates were estimated using a PL 10X/20 eyepiece with graticules (100 mm × 10 mm in 100-grid divisions). The percentage of cells presenting GFP-AR.Q48 cytoplasmic aggregates was divided by the total number of the transfected DsRed-monomer positive cells. At least 150 cells per field were counted, and 3 fields for each coverslip were analyzed. The experiments were performed in triplicate.

### 2.4 Western blot analysis and filter retardation assay

For Western blot (WB) analysis and filter retardation assay (FRA), NSC34 cells were plated in 12-well multiwell plates at 80,000 cells per well and transfected as previously described.

After 48 hours from transfection, cells were harvested and centrifuged for 5 minutes at 100*g* at 4 °C; the cell pellets were resuspended in phosphate buffered saline (added to the protease inhibitor cocktail; Sigma-Aldrich) and homogenized using slight sonication as previously described (Poletti et al., 2001). Total proteins were determined with the bicinchoninic acid method (BCA assay, Euroclone). WB analysis was performed on 15% sodium dodecyl sulfate (SDS)-polyacrylamide gel electrophoresis loading 30 μg of total proteins. Samples were then electrotransferred to PVDF (Polyscreen transfer membrane, PerkinElmer, Watham, MA, USA) using a liquid transfer apparatus (Bio-Rad, Hercules, CA, USA). The membranes were treated with a blocking solution containing 5% nonfat dried milk powder (EuroClone, Pero, Milan, Italy) in TBS-T for 1 hour and then incubated with the primary antibodies: (1) rabbit polyclonal AR-H280 (dilution 1:1000) to detect wild type (wt) AR and ARpolyQ; (2) rabbit polyclonal anti-LC3 (dilution 1:1000) to detect the LC3; (3) rabbit polyclonal anti-SQSTM1/p62 (dilution 1:1000) to detect p62; (4) goat polyclonal anti-actin (Actin I-19; Santa Cruz Biotech, dilution 1:1000) to detect total actin; (5) mouse monoclonal anti-α-tubulin (Sigma-Aldrich, dilution 1:1000) to detect total α-tubulin; (6) home-made rabbit polyclonal anti-HspB8 (kindly provided by Dr Jacques Landry, Quèbec, Canada, dilution 1:1000 in 3% BSA TBS-T); and (7) peroxidase labeled anti-GFP (Vector Laboratories; dilution 1:5000) to detect GFPu. Immunoreactivity was detected using the following secondary peroxidase-conjugated antibodies: goat anti-rabbit (sc-2004, Santa Cruz Biotech, dilution 1:5000) was used to identify the anti-AR, the anti-HspB8, the anti-LC3, and the anti-p62 antibody; donkey anti-goat (sc-2020, Santa Cruz Biotech, dilution 1:5000) was used to identify the anti-actin antibody; goat anti-mouse (sc-2005, Santa Cruz Biotech, dilution 1:5000) was used to identify the anti-α-tubulin antibody. The immunoreactive regions were then visualized using the enhanced chemiluminescence detection kit reagents (ECL prime Western Blotting Substrate, GE Healthcare). The same membranes were subsequently processed with different antibodies to detect the levels of different proteins in the same sample loaded on the gel, after stripping for 10 minutes at room temperature (StripABlot, EuroClone).

FRA was performed using sample filtration through a 0.2-μm cellulose acetate membrane (Whatman, GE Healthcare, Maidstone, UK) using a dot-blot apparatus (Bio-Rad) and loading 1.5 μg of total proteins for AR.Q(n). Dot-blots were probed as described for WB analysis.

A ChemiDoc XRS System (Bio-Rad) was used for the image acquisition of WB and FRA.

Optical intensity of samples assayed with WB or FRA was detected and analyzed using NIH ImageJ software.

### 2.5 Cytofluorimetric analysis

Samples for cytofluorimetric analysis were obtained from NSC34 cells plated in 12-well multiwell plates at a density of 90,000 cells per well. The cells, transfected as described earlier in text, were harvested and centrifuged for 5 minutes at 100*g* at 4 °C. The pellets were resuspended in 300 μL of 4% FBS phosphate buffered saline. Cell fluorescence was detected using FACS Calibur (BD Biosciences, San Jose, CA, USA). Cytofluorimetric results were analyzed using CellQuest (BD Biosciences) analysis software.

### 2.6 Messenger RNA expression analysis

NSC34 were plated at 80,000 cells per mL in 6-well multiwell plates and were transfected as described earlier in text.

Forty-eight hours from transfection, cells were harvested in 4 M guanidium-isothiocyanate (containing 25 mM sodium citrate pH 7.5, 0.5% sarcosyl and 0.1% 2-mercaptoethanol) and total RNA was isolated using phenol-chloroform extraction according to Chomczynski and Sacchi (1987). RNA quantification was carried out by absorption at 260 nm. Total RNA (1 μg) was treated with DNAse and reverse transcribed into cDNA using the High-Capacity cDNA Archive Kit (Applied Biosystems, Life Technologies Corporation, Carlsbad, CA, USA) according to the manufacturer's protocol. Primers for real-time reverse transcription polymerase chain reaction (PCR) of the HspB8, LC3-B, p62, and GAPDH messenger RNAs (mRNAs) were designed in accordance with recommendations accompanying the CFX 96 Real Time System (Bio-Rad) on C-G base content and 5' content and using the program Primer Express 3. The primers were synthesized by MWG Biotech (Ebersberg, Germany) with the following sequence: *mHspB8:* 5'-ATACGTGGAAGTTTCAGGCA-3′ (forward), 5'- TCCTTTGACCTAACGCAACC-3′ (reverse); *mMAP-LC3b*: 5′- CGT CCT GGA CAA GAC CA-3′ (forward), 5′-CCA TTC ACC AGG AGG AA-3′ (reverse); *mp62:* 5′-AGG GAA CAC AGC AAG CT-3′ (forward), 5′-GCC AAA GTG TCC ATG TTT CA-3′ (reverse); *mGAPDH*: 5′-CCA GAA CAT CAT CCC TGC AT -3′ (forward), 5′-CAG TGA GCT TCC CGT TCA-3′ (reverse). The evaluated efficiency of each set of primers was close to 100% for target and reference genes. Real-time PCR was performed using the CFX 96 Real Time System (Bio-Rad) in 10 μL total volume, using the iTaq SYBR Green Supermix (Bio-Rad), and with 500 nM primers. PCR cycling conditions were as follows: 94 °C for 10 minutes, 35 cycles at 94 °C for 15 seconds and 60 °C for 1 minute. Melting curve analysis was always performed at the end of each PCR assay as a control for specificity. Data were expressed as Ct values and used for the relative quantification of targets using the ΔΔCt calculation. To exclude potential bias because of averaging data were transformed using the equation 2^−ΔΔCt^ to give N-fold changes in gene expression; all statistics were performed with ΔCt values. Each experiment was carried out using 4 independent samples. HspB8, LC3, and p62 values were then normalized with those of GAPDH.

### 2.7 Transcriptional activity

For promB8 transcriptional activity, cells were plated at 80,000 cells per mL in 24-well multiwell plates and were transfected with promB8 and pRL-TK plasmids as described earlier in text. Starting from the day of transfection, cells were treated with trehalose 100 mM. Forty-eight hours after transfection, transcriptional assay was performed using the Dual-Glo Luciferase Assay System (Promega) as previously described (Crippa et al., 2010b).

### 2.8 Statistical analysis

Statistical analysis was performed using 1-tailed Student *t* test for 2-group comparisons and 2-way ANOVA for 3 or more group comparisons using PRISM software, version 5.0a (GraphPad Software, La Jolla, CA, USA).

## 3 Results

To determine whether HspB8 counteracts ARpolyQ accumulation by facilitating its clearance and whether a possible link with testosterone AR activation might occur, we evaluated the levels of soluble and aggregated forms of ARpolyQ after HspB8 overexpression.

### 3.1 Effect of HspB8 on ARpolyQ biochemical behavior during testosterone activation

**Fig. 1:**
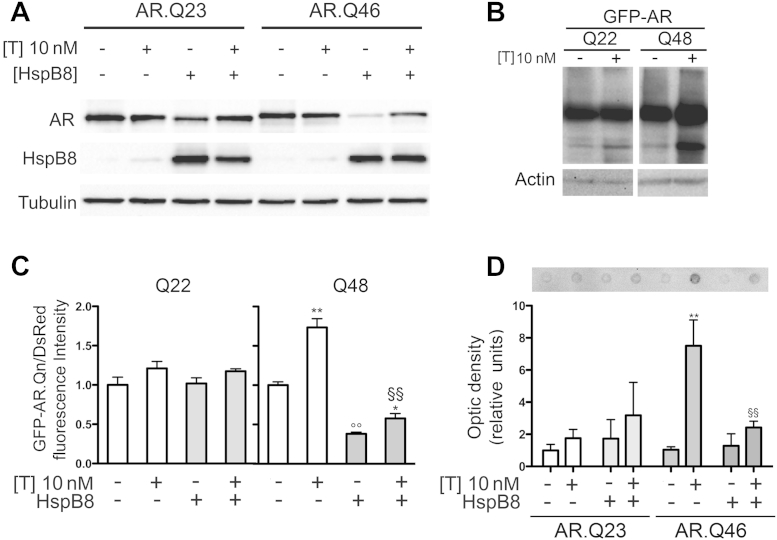
Effect of heat shock protein (Hsp)B8 on wild type (wt) and mutant androgen receptor (AR) gene with an abnormally long polyglutamine tract (ARpolyQ) aggregation and clearance. (A) Western blot analysis on cell lysates of NSC34 expressing wt AR (AR.Q23) or mutant ARpolyQ (AR.Q46), pCDNA3 or HspB8 in the absence (−T) or in the presence (+T) of 10 nM of T for 48 hours. α-tubulin was used to normalize protein loading. (B) Western blot analysis performed on NSC34 cells transfected with GFP-AR.Q22 or GFP-AR.Q48, in the absence (−T) or in the presence (+T) of 10 nM of T for 48 hours. β-actin was used to normalize protein loading. (C) Cytofluorimetric analysis performed on NSC34 expressing DsRed monomer, GFP-AR.Q22, or GFP-AR.Q48, pCDNA3, or HspB8 in the absence (−T) or in the presence (+T) of 10 nM of T for 48 hours (* *p* < 0.05 vs. T-untreated controls; ** *p* < 0.01 vs. T-untreated controls; °° *p* < 0.01 vs. GFP-AR.Q48−T; §§ *p* < 0.01 vs. GFP-AR.Q48+T). (D) Filter retardation assay performed on NSC34 cells transfected with AR.Q23 or AR.Q46, pCDNA3, or HspB8 in the absence (−T) or in the presence (+T) of 10 nM of T for 48 hours (** *p* < 0.01 vs. T-untreated controls; §§ *p* < 0.01 vs. AR.Q46+T). Abbreviations: T, testosterone; pCDNA3, empty vector.

We have previously shown that HspB8 overexpression increases the clearance of mutant ARpolyQ in cells that are not treated with its ligand, testosterone (Carra et al., 2005). However, the aggregation properties of ARpolyQ change depending on the absence or presence of testosterone. We thus measured the levels of soluble and aggregated forms of ARpolyQ in cells overexpressing HspB8 either in absence or presence of testosterone. As shown in Fig. 1A, in our immortalized motoneuronal model of SBMA, HspB8 overexpression reduced the levels of monomeric SDS-soluble ARpolyQ (AR.Q46) in the absence and presence of testosterone, and the effect of HspB8 on wt AR (AR.Q23) was negligible. The ARpolyQ can exist as SDS-soluble monomers, higher molecular weight (HMW) homo- and/or heteromers and aggregates (Merry et al., 1998), the latter being not readily detectable using WB analysis. In addition, low but significant amounts of fragmented ARs can also be formed (Merry et al., 1998), hardly quantifiable in WB (unless WB is overexposed, see an example in Fig. 1B; see also WB obtained with untagged ARpolyQ, later in text). We thus developed a cytofluorimetric assay to quantify the effect of HspB8 on different ARpolyQ forms, measuring all chimeric fragmented GFP-AR (N-terminally tagged GFP-AR fragments) and full-length species containing the polyQ and including also insoluble material. Fig. 1C shows that though testosterone treatment did not affect the levels of wt AR (GFP-AR.Q22), it leads to a strong accumulation of the mutant ARpolyQ form (GFP-AR.Q48), which was significantly reverted by the coexpression of HspB8.

The strong effect exerted by HspB8 specifically on the mutant ARpolyQ might reveal a specificity of HspB8 to aggregate-prone species by recognizing and clearing both and/or either HMW and/or aggregated AR species. In fact, inclusions formation is a complex and dynamic multistep process. Soluble misfolded species must interact together to form intermediate and insoluble species. At a specific time point, soon after testosterone-induced activation (and release from protective Hsps) these species exist in a “soluble and dynamic,” but already aggregated status (Simeoni et al., 2000; Stenoien et al., 1999, 2002), and are believed to protect against mutant protein toxicity (Rusmini et al., 2007, 2011). Indeed, using FRA, in which HMW ARpolyQ species can be retained and quantified, we showed that HspB8 significantly and efficiently reduced the total amount of ARpolyQ (AR.Q46) insoluble species formed after testosterone treatment (Fig. 1D). To confirm the specificity of the HspB8 prodegradative effects on misfolded ARpolyQ, we downregulated HspB8 expression with a specific siRNA and, using FRA, quantified the levels of misfolded ARpolyQ-insoluble species. HspB8 silencing correlated with a robust accumulation of testosterone-treated ARpolyQ insoluble species (see below), while, as expected, the control scramble siRNA had no effect on insoluble ARpolyQ. Thus, even if HspB8 basal expression levels are low in NSC34 cells (Crippa et al., 2010b), this endogenous concentration of HspB8 is already sufficient to remove a large relevant, but not the entire fraction, of misfolded ARpolyQ-insoluble species present in these cells. This provides an explanation for the ARpolyQ accumulation into aggregates after testosterone treatment. The higher protein levels achievable with exogenous HspB8 overexpression, thus allow this chaperone to fully clear misfolded ARpolyQ-insoluble species, preventing ARpolyQ aggregation.

**Fig. 2:**
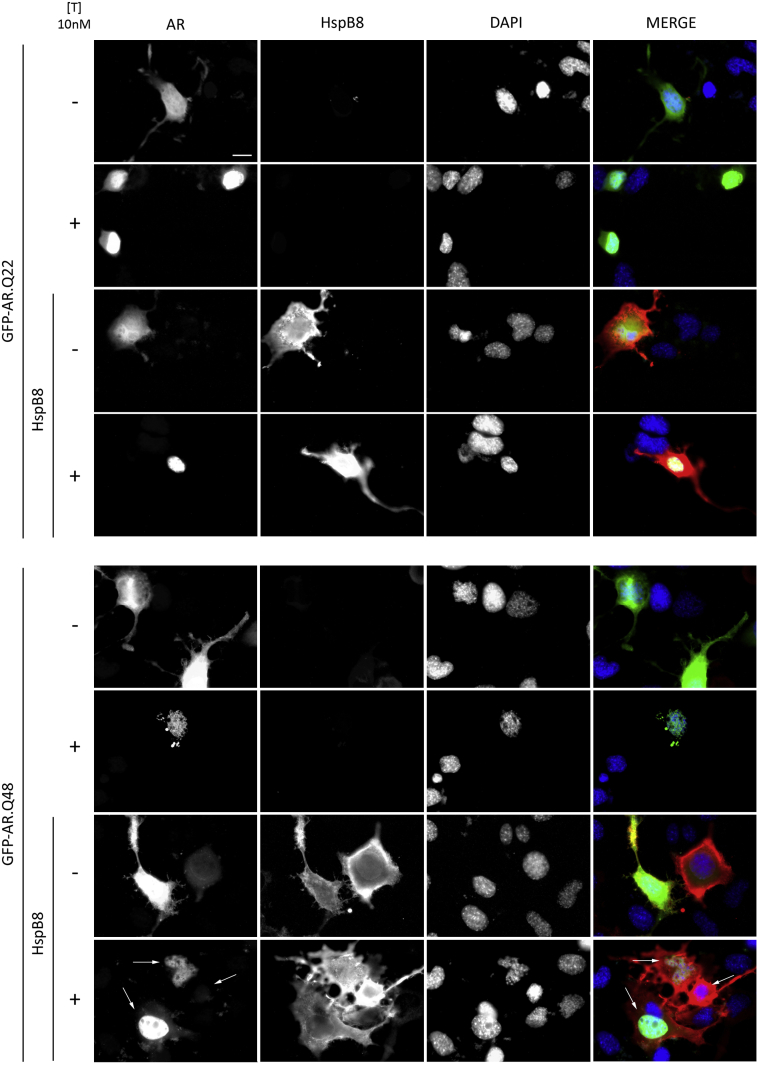
Effect of heat shock protein (Hsp)B8 on aggregation and intracellular distribution of wild type (wt) and mutant androgen receptor (AR) gene with an abnormally long polyglutamine tract (ARpolyQ). High resolution immunofluorescence microscopy analysis (magnification ×63) on NSC34 cells transfected with GFP-AR.Q22 or GFP-ARQ.48 (green), pCDNA3 or HspB8 (red) in the absence (−T) or in the presence (+T) of 10 nM of T for 48 hours. Nuclei were stained with DAPI (blue) (scale bar, 10 μm). Abbreviations: DAPI, 4′,6-diamidino-2-phenylindole; pCDNA3, empty vector; T, testosterone.

We also analyzed, using “high resolution fluorescence microscopy,” the intracellular distribution of wt and mutant AR in the absence and presence of testosterone and/or HspB8. Though wt AR (GFP-AR.Q22) showed no aggregates either in the absence or presence of testosterone, testosterone treatment induced cytoplasmic ARpolyQ inclusions (GFP-AR.Q48) in 12.69% (± 0.96) of our immortalized motoneurons (Fig. 2) (Rusmini et al., 2007, 2011; Simeoni et al., 2000). HspB8 reduced aggregate formation, and only 6.98% (± 0.72; *p* < 0.01) of transfected cells were aggregate-positive (Fig. 2) in line with the data obtained with FRA on insoluble ARpolyQ (Fig. 1D). Interestingly, an inverse correlation was noted between HspB8 expression levels and ARpolyQ accumulation, suggestive of an increased HspB8-mediated clearance of GFP-AR.Q48 (see arrows in the lower insets of Fig. 2). Notably, HspB8 had no effect on wt AR (GFP-AR.Q22), supporting the notion that HspB8 only mildly affects the non-pathological and non-aggregate-prone AR forms (either ARpolyQ non-activated with testosterone or the wt AR in any condition), and significantly inhibits the aggregation and accumulation of the pathologic ones.

### 3.2 Effect of HspB8 on ARpolyQ-induced alterations of the proteasome pathway

**Fig. 3:**
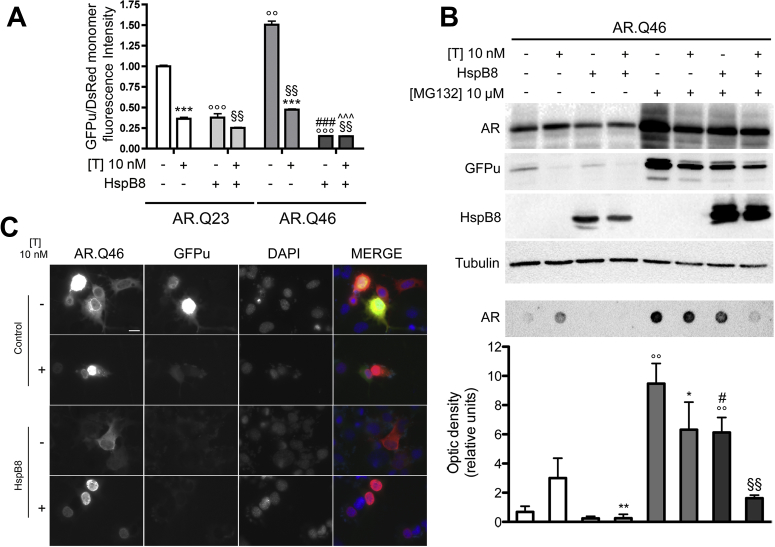
.The antiaggregation/prodegradative activity of heat shock protein (Hsp)B8 on androgen receptor (AR) gene with an abnormally long polyglutamine tract (ARpolyQ) does not require the ubiquitin proteasome system. (A) Cytofluorimetric analysis performed on NSC34 cells expressing GFPu, DsRed monomer, AR.Q23 or AR.Q46, pCDNA3, or HspB8 in the absence (−T) or in the presence (+T) of 10 nM of T for 48 hours (*** *p* < 0.001 vs. T-untreated controls; °°° *p* < 0.001 vs. AR.Q23−T; §§ *p* < 0.01 vs. AR.Q23+T; ### *p* < 0.001 vs. AR.Q46−T; ˆˆˆ *p* < 0.001 vs. AR.Q46+T; °° *p* < 0.01 vs. AR.Q23−T). (B) Upper insets, Western blot analysis, and lower insets, filter retardation assay, on cell lysates of NSC34 cells expressing GFPu, AR.Q46, pCDNA3, or HspB8 in the absence (−T) or in the presence (+T) of 10 nM of T for 48 hours, in basal condition or after treatment with 10 μM of MG132 for 24 hours. α-Tubulin was used to normalize protein loading. (°° *p* < 0.01 vs. T-untreated controls; ** *p* < 0.01 vs. T-treated controls; * *p* < 0.05 vs. T-treated controls; # *p* < 0.05 vs. −T/+MG132; §§ *p* < 0.01 vs. +T/+MG132). (C) High resolution immunofluorescence analysis of NSC34 cells transfected with GFPu, AR.Q46, pCDNA3, or HspB8 in the absence (−T) or in the presence (+T) of 10 nM of T for 48 hours. Nuclei were stained with DAPI (blue). Images were obtained at magnification ×63 (scale bar, 10 μm). Abbreviations: DAPI, 4,6-diamidino-2-phenylindole; GFPu, green fluorescent protein fused to a constitutive degron signal, CL1; MG132, Z-leu-leu-leu-al; pCDNA3, empty vector; T, testosterone.

As mentioned, our published data demonstrated that soluble (testosterone-untreated) ARpolyQ overwhelms UPS activity, and aggregated ARpolyQ (testosterone-treated) allows UPS desaturation (Rusmini et al., 2007). Thus HspB8, by preventing ARpolyQ aggregation, could indirectly increase the pool of soluble ARpolyQ species that are routed to UPS degradation and could overwhelm it. To test this hypothesis, UPS function was evaluated in the absence and presence of HspB8 by monitoring the degradation of the UPS reporter protein GFPu (Bence et al., 2001), which accumulates when UPS is impaired (Rusmini et al., 2007; Sau et al., 2007). The cytofluorimetric assay confirmed that (Fig. 3A) GFPu accumulated only in motoneuronal cells expressing soluble ARpolyQ (AR.Q46), and testosterone-induced ARpolyQ aggregation correlated with increased GFPu clearance, as an index of restored UPS activity. HspB8 coexpression with ARpolyQ (with or without testosterone) always correlated with increased GFPu removal from motoneuronal cells (Fig. 3A) demonstrating that the antiaggregation activity of HspB8 on ARpolyQ does not alter UPS functions.

WB (Fig. 3B, upper insets) and immunofluorescence (Fig. 3C) analyses confirmed that GFPu accumulates in basal condition. Indeed, analyzing UPS activity in single cells expressing ARpolyQ (soluble or aggregated), we found that cells in which testosterone-activated ARpolyQ was still present did not show aggregates indicating a pronounced clearance mediated by HspB8; here, no GFPu accumulation was detectable (Fig. 3C).

In addition, we investigated the effects of HspB8 when UPS activity is pharmacologically inhibited by the proteasome inhibitor, MG132. In FRA, we found that even when UPS is inactivated, HspB8 greatly reduced (even if not completely) the accumulation of misfolded ARpolyQ-insoluble species (Fig. 3B, lower insets). Notably, we found an inverse stabilization of ARpolyQ after testosterone treatment with or without UPS inhibition. Indeed, as shown in WB analysis, with unblocked UPS, testosterone increased ARpolyQ levels (Piccioni et al., 2002; Rusmini et al., 2007, 2010, 2011; Simeoni et al., 2000). When the UPS is inhibited, untreated ARpolyQ accumulated at very high levels, confirming its preferential degradation via UPS. Conversely, the testosterone-treated ARpolyQ (mainly composed of misfolded insoluble species) does not undergo the same increase and/or stabilization, possibly because misfolded ARpolyQ uses alternative degradative pathways, such as the autophagic system. However, basal autophagy does not completely remove ARpolyQ, which accumulates into intracellular aggregates. Because misfolded ARpolyQ insoluble species almost completely disappear in the presence of HspB8 when UPS is blocked, we evaluated the possibility that the HspB8 prodegradative activity has to be due to autophagy.

### 3.3 Involvement of the autophagic pathway in the HspB8-mediated clearance of ARpolyQ

**Fig. 4:**
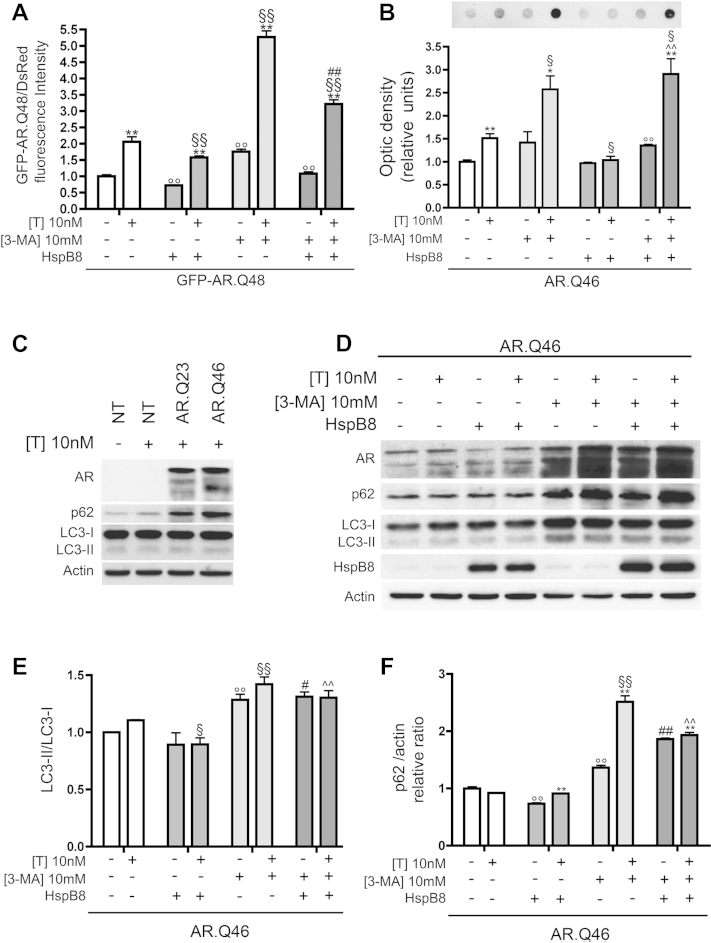
Active autophagy is required to mediate the antiaggregation and/or prodegradative activity of heat shock protein (Hsp)B8 on the androgen receptor (AR) gene with an abnormally long polyglutamine tract (ARpolyQ). (A) Cytofluorimetric analysis performed on NSC34 cells expressing DsRed monomer, GFP-AR.Q48, pCDNA3, or HspB8 in the absence (−T) or in the presence (+T) of 10 nM of T for 48 hours, in basal condition or after treatment with 10 mM of 3-methyladenine (3-MA) for 24 hours (** *p* < 0.01 vs. T-untreated controls; °° *p* < 0.01 vs. GFP-AR.Q48−T; §§ *p* < 0.01 vs. GFP-AR.Q48+T; ## *p* < 0.01 vs. GFP-AR.Q48+T+HspB8). (B) Filter retardation assay performed on NSC34 cells transfected with AR.Q46, pCDNA3, or HspB8 in the absence (−T) or in the presence (+T) of 10 nM of T for 48 hours, in basal condition or after treatment with 10 mM of 3-MA for 24 hours (* *p* < 0.05 vs. T-untreated controls; ** *p* < 0.01 vs. T-untreated controls; °° *p* < 0.01 vs. AR.Q46−T; § *p* < 0.05 vs. AR.Q46+T; ˆˆ *p* < 0.01 vs. AR.Q46+T+HspB8). (C) Western blot analysis on cell lysates of NSC34 cells untrasfected (NT) or expressing AR.Q23 or AR.Q46, in the absence (−T) or in the presence (+T) of 10 nM of T for 48 hours. β-actin was used to normalize protein loading. (D) Western blot analysis on cell lysates of NSC34 cells expressing AR.Q46, pCDNA3 or HspB8 in the absence (−T) or in the presence (+T) of 10 nM of T for 48 hours, in basal condition or after treatment with 10 mM of 3-MA for 24 hours. β-actin was used to normalize protein loading. (E) The histogram represents a quantitative evaluation of LC3-II/LC3-I ratio protein level carried out by densitometric scanning of the blots (3 replicates) (°° *p* < 0.01 vs. AR.Q46−T; § < 0.05 vs. AR.Q46+T; §§ *p* < 0.01 vs. AR.Q46+T; # *p* < 0.05 vs. AR.Q46−T+HspB8; ˆˆ *p* < 0.005 vs. AR.Q46+T+HspB8). (F) The histogram represents a quantitative evaluation of p62 protein level normalized on actin carried out by densitometric scanning of the blots (3 replicates) (°° *p* < 0.01 vs. AR.Q46−T; §§ *p* < 0.01 vs. AR.Q46+T; ** *p* < 0.01 vs. T-untreated controls; ## *p* < 0.01 vs. AR.Q46−T+HspB8; ˆˆ *p* < 0.01 vs. AR.Q46+T+HspB8). Abbreviations: pCDNA3, empty vector; T, testosterone.

The UPS is typically counterbalanced by the autophagic system. A regulatory cross-talk exists between the two systems, to maintain a proper balance in intracellular protein homeostasis (Gamerdinger et al., 2011a, 2011b). We have shown that UPS is impaired by the expression of ARpolyQ per se. As a consequence of UPS impairment, cells expressing mutant ARpolyQ would need to preferentially target it to autophagy degradation. HspB8 overexpression might thus assist in this autophagy-targeting step. In fact, we previously published that HspB8, in association with the cochaperone BAG-3, leads to autophagy-mediated degradation of other aggregate-prone misfolded proteins (Carra et al., 2008a, 2008b; Crippa et al., 2010a, 2010b). Using cytofluorimetric analysis we studied the effect of HspB8 on the autophagy-mediated clearance of GFP-AR.Q48 (normalized by dsRED monomer). We first confirmed that ARpolyQ is degraded by autophagy (Fig. 4A). As expected, treatment with 3-MA, an autophagy inhibitor that prevents autophagosome formation at very early stages (Klionsky et al., 2012; Rubinsztein et al., 2009), led to the accumulation of a large ARpolyQ fraction (in the presence or absence of testosterone treatment). Similarly, accumulation of mutant ARpolyQ was observed in cells expressing HspB8 and treated with 3-MA, showing that HspB8 requires autophagy to exert its antiaggregation and/or prodegradative effects. These data were also in line with the data obtained using FRA (Fig. 4B), which demonstrated that autophagy inhibition correlated with a lack of HspB8 effect on the clearance of testosterone-induced misfolded ARpolyQ-insoluble species retained by the cellulose acetate membrane.

We next analyzed whether and/or how HspB8 modulates the autophagy system to remove aggregates from motoneuronal cells. We evaluated autophagy activation using two well-known markers: LC3 and p62 (Klionsky et al., 2012). LC3 exists in two forms: LC3-I, which has a diffuse cytoplasmic distribution, and lipidated LC3-II, which is recruited to the membrane of the autophagosome allowing their formation (Klionsky et al., 2012). p62 is a protein that recognizes and/or binds to (poly)ubiquitinated substrates and also interacts with LC3, thus allowing substrate targeting to autophagosomes (Klionsky et al., 2012). Both proteins are overexpressed during autophagy activation and accumulate when the autophagic flux is impaired (Klionsky et al., 2012; Rubinsztein et al., 2009). Moreover, their protein levels are tightly transcriptionally controlled, thus their rate of synthesis is influenced by the rate of their clearance via autophagy. In particular, when autophagic flux is insufficient (or impaired), p62 (normally diffused into the cytoplasm) accumulates into the so called “p62 bodies” which are capable of triggering aberrant pathways inside the cells (Matsumoto et al., 2011). To monitor the autophagic process we thus analyzed LC3 and p62 mRNA and protein levels and their subcellular distribution in cells expressing mutant ARpolyQ and/or HspB8, in the absence and presence of specific autophagy inhibitors (Klionsky et al., 2012). We first analyzed LC3-I to LC3-II conversion and p62 levels in NSC34 cells not expressing AR (either wt or mutant). As shown in Fig. 4C, we failed to detect p62 accumulation or LC3 activation either in the absence or presence of testosterone, indicating that in our cell model, the autophagy process is not basically activated. However, a mild increase of p62 was present in NSC34 cells expressing wt AR (a protein with a slight tendency to misfold), and a robust increase of p62 was detectable in NSC34 cells expressing mutant ARpolyQ (which has a great tendency to misfold), paralleled by a slight increase in LC3-II levels. We next evaluated the possible effect of HspB8 on autophagic flux. We followed endogenous LC3-II/LC3-I dynamic and p62 levels, particularly when autophagy was pharmacologically blocked with 3-MA, because LC3-II and p62 usually undergo a rapid degradation via autophagy. The WB (Fig. 4D) indicates that endogenous LC3-I levels remained substantially unchanged in all basal conditions tested. As expected, an increase of LC3-I was evident during autophagy flux blockage with 3-MA. This 3–MA-dependent increase in LC3-I levels is related to inhibition of its turnover via autophagolysosomes. Interestingly, in testosterone-treated NSC34 cells expressing ARpolyQ, HspB8 overexpression resulted in a slight, but significant reduction of LC3-II/LC3-I ratio when compared with HspB8 untransfected cells (see quantification in Fig. 4E); no significant variations were observed in the case of testosterone-untreated cells (no ARpolyQ aggregation). In the presence of 3-MA, which inhibits autophagosome formation thus inducing LC3-II accumulation, we found no effects of HspB8 on the LC3-II/LC3-I ratio (Fig. 4E).

Because increased LC3-II levels observed on ARpolyQ overexpression could be because of flux blockage (Klionsky et al., 2012), rather than increased production of LC3 (Klionsky et al., 2012; Onesto et al., 2011), we also analyzed variations on p62 dynamic induced by mutant ARpolyQ and the effects of HspB8 on these parameters. Notably, p62 levels were found robustly increased in the presence of mutant ARpolyQ and 3-MA (Fig. 4D, and quantification in Fig. 4F). As expected, in the absence of HspB8, autophagy blockage resulted in a larger increase of p62 accumulation in cells treated with testosterone, when aggregates are generated; in fact, in absence of testosterone, ARpolyQ is soluble and mainly processed via the UPS (in agreement with the data presented in Fig. 3), thus p62 levels are less affected. In the case of HspB8 overexpression, we found a slight decrease in p62 levels with or without testosterone, suggesting that HspB8 seems to reroute p62 degradation via UPS.

As mentioned earlier in text, steady-state LC3 and p62 levels and LC3-II/LC3-I ratio are consequence of the final equilibrium of protein synthesis and clearance, which are both affected by autophagy activation and autophagy flux blockage. Thus, to corroborate our working hypothesis of an HspB8 proautophagic degradative activity on mutated ARpolyQ, we further analyzed the localization of the exogenous LC3 and p62 in our experimental conditions (according to the widely accepted guidelines on autophagy markers usage; Klionsky et al., 2012).

**Fig. 5:**
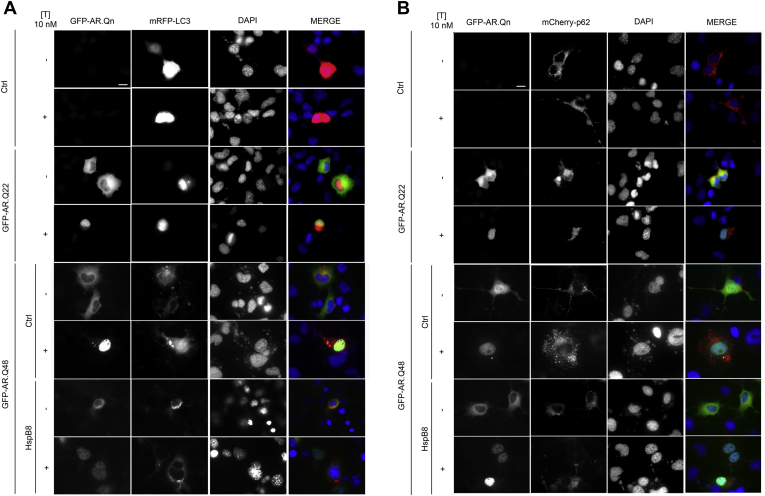
Autophagy is activated by the presence of androgen receptor (AR) gene with an abnormally long polyglutamine tract (ARpolyQ), but not by heat shock protein (Hsp)B8. (A) High-resolution fluorescence microscopy analysis (magnification ×63) on NSC34 cells expressing mRFP-LC3, and pCDNA3, GFP-AR.Q22 or GFP-AR.Q48 (−/+HspB8), in the absence (−T) or in the presence (+T) of 10 nM of T. Nuclei were stained with DAPI (blue) (scale bar, 10 μm). (B) High-resolution fluorescence microscopy analysis (magnification ×63) on NSC34 cells expressing mCherry-p62 and pCDNA3, GFP-AR.Q22 or GFP-AR.Q48 (−/+HspB8) in the absence (−T) or in the presence (+T) of 10 nM of T. Nuclei were stained with DAPI (blue) (scale bar, 10 μm). Abbreviations: Ctrl, control; DAPI, 4′,6-diamidino-2-phenylindole; GFP, green fluorescent protein; mRFP-LC3, monomeric red fluorescent protein; pCDNA3, empty vector; T, testosterone.

The analysis of exogenous LC3 (mRFP-LC3) intracellular distribution is shown in Fig. 5A. It appears that, in nontransfected NSC34 cells (either with or without testosterone) autophagy is not activated; in fact exogenous mRFP-LC3 shows a diffused intracellular distribution (in line with WB data shown in Fig. 4C). A slight autophagy activation could be observed in NSC34 cells expressing wt GFP-AR.Q22 (either with or without testosterone), because mRFP-LC3 clearance appears higher than that measured in the absence of AR protein (as already mentioned, it is very well known that even wt AR has a tendency to generate few misfolded species (Marcelli et al., 2006; Palazzolo et al., 2008; Piccioni et al., 2001, 2002; Poletti, 2004; Rusmini et al., 2007, 2010, 2011; Simeoni et al., 2000; Stenoien et al., 1999)), but most of the remaining LC3 protein still presents a diffuse distribution. Interestingly, a very similar condition could be found in NSC34 cells expressing a mutant form of ARpolyQ (GFP-AR.Q48) in the absence of testosterone, in its nontoxic conformations. On the contrary, ARpolyQ treated with testosterone, (neurotoxic and/or aggregated species), induces a marked alteration of intracellular mRFP-LC3 localization, which becomes clearly detectable in its punctate distribution (lipidated LC3-II, indicative of autophagy activation, but not necessarily of autophagic flux completion). However, in these experiments (and the other performed analyzing the endogenous LC3-II, see below) we observed neither LC3-II/ARpolyQ colocalization nor LC3-II sequestration into ARpolyQ aggregates. This demonstrates that aggregated ARpolyQ is not entrapped in newly formed autophagosomes (not yet fused to lysosome), thus suggesting an impairment of its recruitment within these structures.

The same analysis was performed on p62. Fig. 5B shows the distribution of exogenous mCherry-p62 in NSC34 under various conditions. Testosterone alone has no effect on p62 levels and distribution (in line with WB data shown in Fig. 4C). Only a minor increase of p62 fluorescence, but no alteration in distribution, was observed in NSC34 cells expressing wt AR (GFP-AR.Q22, either in the absence or presence of testosterone). In contrast, and in line with the data obtained with LC3, a marked p62 accumulation was evident in cells expressing mutant ARpolyQ (GFP-AR.Q48), particularly in those treated with testosterone. Notably, p62 tends to generate a large number of large p62 bodies, which often surrounded the ARpolyQ aggregates present in our motoneuronal cells. Finally, the data in Fig. 5B show that p62 accumulation (and ARpolyQ aggregation) can be counteracted by HspB8 expression, in line with the data obtained on LC3-II formation (see Fig. 5A).

**Fig. 6:**
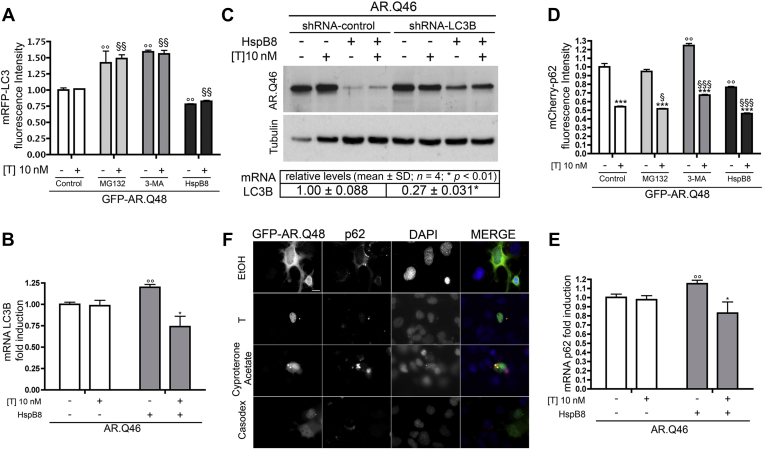
Autophagic flux is impaired by the mutant androgen receptor (AR) gene with an abnormally long polyglutamine tract (ARpolyQ) and restored by heat shock protein (Hsp)B8. (A) Cytofluorimetric analysis performed on NSC34 cells expressing mRFP-LC3, GFP-AR.Q48, pCDNA3, or HspB8 in the absence (−T) or in the presence (+T) of 10 nM of T for 48 hours, in basal condition or after treatment with 10 μM of MG132 or 10 mM of 3-methyladenine (3-MA) for 24 hours (°° *p* < 0.01 vs. GFP-AR.Q48−T; §§ *p* < 0.01 vs. GFP-AR.Q48+T). (B) Real-time polymerase chain reaction (PCR) on LC3B mRNA expression levels on NSC34 cells expressing AR.Q46, pCDNA3, or HspB8 in the absence (−T) or in the presence (+T) of 10 nM of T for 48 hours (°° *p* < 0.01 vs. AR.Q46−T; * *p* < 0.05 vs. AR.Q46+T). (C) Top insets, Western blot analysis of cell lysates of NSC34 cells expressing AR.Q46, pCDNA3, or HspB8 and shRNA against LC3B or shRNA scrambled control in the absence (−T) or in the presence (+T) of 10 nM of T for 48 hours. α-Tubulin was used to normalize protein loading. Bottom inset, real time PCR on LC3B mRNA levels on NSC34 cells expressing shRNA-LC3B or shRNA-control. Data (expressed as fold changes) have been normalized to the amount of GAPDH mRNA, and are expressed as relative to the levels determined in shRNA-control transfected cells, which are taken as internal reference. Data are mean ± SD of 4 independent replicates. (D) Cytofluorimetric analysis performed on NSC34 expressing mCherry-p62, GFP-AR.Q48, pCDNA3, or HspB8 in the absence (−T) or in the presence (+T) of 10 nM of T for 48 hours, in basal condition or after treatment with 10 μM of MG132 or 10 mM of 3-MA for 24 hours (*** *p* < 0.001 vs. T-untreated controls; °° *p* < 0.01 vs. GFP-AR.Q48−T; § *p* < 0.05 vs. GFP-AR.Q48+T; §§§ *p* < 0.001 vs. GFP-AR.Q48+T). (E) Real-time PCR on p62 mRNA expression levels on NSC34 cells expressing AR.Q46, pCDNA3, or HspB8 in the absence (−T) or in the presence (+T) of 10 nM of T for 48 hours (°° *p* < 0.01 vs. AR.Q46−T; * *p* < 0.05 vs. AR.Q46−T+HspB8). (F) High resolution immunofluorescence analysis on NSC34 cells transfected with GFP-AR.Q48 in the absence (−T) or in the presence (+T) of 10 nM of T or 100 nM of cyproterone acetate or 100 nM of casodex for 48 hours. Nuclei were stained with DAPI (blue). Images were obtained at magnification ×63 (scale bar, 10 μm). Abbreviations: DAPI, 4′,6-diamidino-2-phenylindole; GAPDH, glyceraldehyde-3-phosphate dehydrogenase; MG132, Z-leu-leu-leu-al; mRFP-LC3, monomeric red fluorescent protein-LC3; mRNA, messenger RNA; pCDNA3, empty vector; shRNA, short hairpin RNA; T, testosterone.

**Fig. 7:**
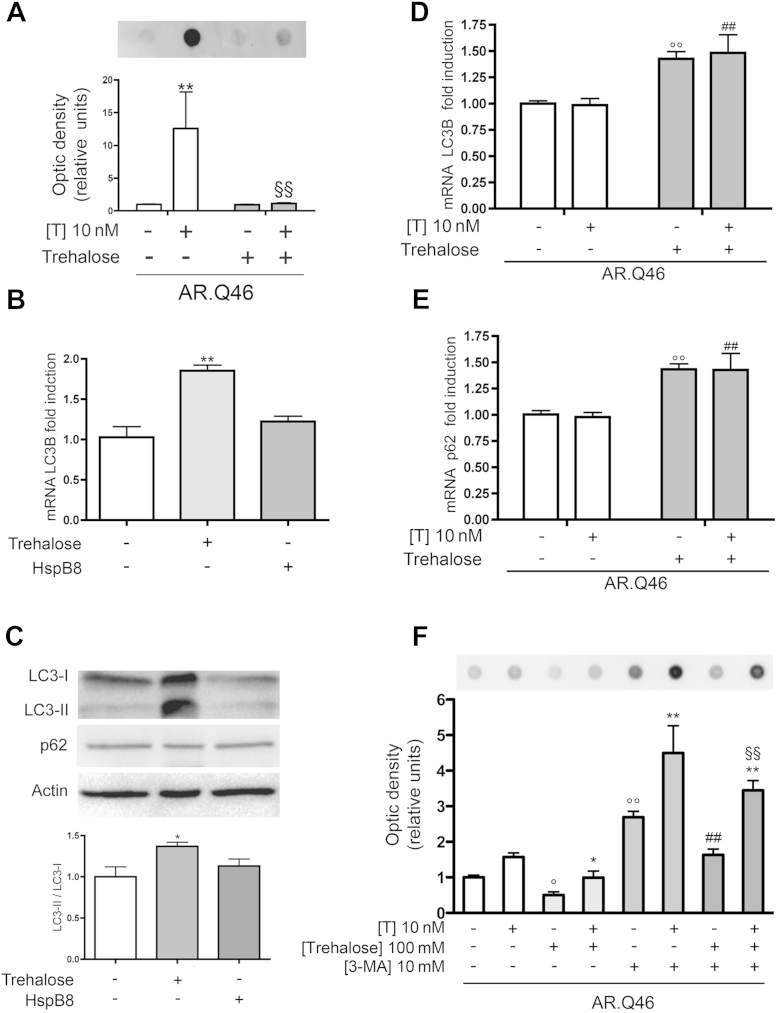
Trehalose induces the clearance of mutant androgen receptor (AR) gene with an abnormally long polyglutamine tract (ARpolyQ), via autophagy activation. (A) Filter retardation assay performed on NSC34 cells transfected with AR.Q46 in the absence (−T) or in the presence (+T) of 10 nM of T for 48 hours, in basal condition or after treatment with 100 mM of trehalose for 48 hours (** *p* < 0.01 vs. T-untreated control; §§ *p* < 0.01 vs. AR.Q46+T). (B) Real-time polymerase chain reaction (PCR) on LC3B mRNA expression levels on NSC34 cells expressing pCDNA3 or heat shock protein (Hsp)B8 in basal condition or after treatment with 100 mM of trehalose for 48 hours (** *p* < 0.01 vs. untreated control). (C) Upper insets, Western blot analysis of cell lysates of NSC34 expressing pCDNA3 or HspB8 in basal condition or after treatment with 100 mM of trehalose for 48 hours. β-actin was used to normalize protein loading. Lower inset, the histogram represents a quantitative evaluation of LC3-II/LC3-I ratio protein level carried out by densitometric scanning of the blots (3 replicates) (* *p* < 0.05 vs. untreated control). (D) Real-time PCR on LC3B mRNA expression levels on NSC34 cells expressing AR.Q46 in the absence (−T) or in the presence (+T) of 10 nM of T for 48 hours, in basal condition or after treatment with 100 mM of trehalose for 48 hours (°° *p* < 0.01 vs. AR.Q46−T; ## *p* < 0.01 vs. AR.Q46+T). (E) Real-time PCR of p62 mRNA expression levels on NSC34 cells expressing AR.Q46 in the absence (−T) or in the presence (+T) of 10 nM of T for 48 hours, in basal condition or after the treatment with 100 mM of trehalose for 48 hours (°° *p* < 0.01 vs. AR.Q46−T; ## *p* < 0.01 vs. AR.Q46+T). (F) Filter retardation assay performed on cell lysates of NSC34 cells expressing AR.Q46, in the absence (−T) or in the presence (+T) of 10 nM of T for 48 hours, in basal condition or after treatment with 10 mM of 3-MA or 100 mM of trehalose for 24 hours. (° *p* < 0.05 and °° *p* < 0.01 vs. AR.Q46−T; * *p* < 0.05 and ** *p* < 0.01 vs. AR.Q46+T; ## *p* < 0.01 vs. AR.Q46−T/+trehalose; §§ *p* < 0.01 vs. AR.Q46+T/+trehalose). Abbreviations: mRNA, messenger RNA; pCDNA3, empty vector; T, testosterone.

In light of these data we wanted to further unravel the mechanism of action of HspB8. For this purpose, we deeply analyzed the intracellular regulation of LC3 and p62. Fig. 6A shows a cytofluorimetric analysis that clearly demonstrates a robust LC3 accumulation when proteasome or autophagy are pharmacologically blocked with selective inhibitors. By contrast, exogenous LC3 levels significantly decreased in motoneuronal cells coexpressing mutant ARpolyQ and HspB8. To discriminate between LC3 variation because of (1) its de novo transcription; and (2) its clearance (Klionsky et al., 2012; Rubinsztein et al., 2009), we also evaluated whether LC3 transcription is induced in the presence of ARpolyQ and/or HspB8. In fact, LC3 expression usually increases when autophagy needs to be upregulated (i.e., autophagic flux blockage). Surprisingly, LC3 mRNA levels were similar in NSC34 cells expressing ARpolyQ either in absence or presence of testosterone (Fig. 6B), suggesting that the UPS function modifications that occur in the presence of ARpolyQ with or without testosterone (see Fig. 3) are not sufficient to affect LC3 expression. Instead, after coexpression of HspB8, we found that in NSC34 cells expressing soluble, nonactivated ARpolyQ (AR.Q46 without testosterone, in which UPS functions are overwhelmed; see Fig. 3A) LC3 transcription was increased. Conversely, in NSC34 cells in which ARpolyQ tends to aggregate (testosterone-activated AR.Q46 and normal UPS activity), HspB8 not only prevented ARpolyQ aggregation, but significantly reduced LC3 mRNA expression to levels lower than that found in its testosterone-untreated control and in the absence of HspB8 (Fig. 6B). Interestingly, HspB8 alone does not induce LC3 mRNA expression (see later in text, and Fig. 7B and C). Thus, when misfolded ARpolyQ fraction cannot accumulate and is rapidly cleared from motoneuronal cells (see Fig. 1), the autophagic flux proceeds to completion, and LC3 levels decrease. This further supports the notion that HspB8 acts by ameliorating the autophagic removal of misfolded proteins rather than by directly activating autophagy (Carra et al., 2012; Crippa et al., 2010a, 2010b).

Together these data on endogenous LC3 mRNA levels and exogenous mRFP-LC3 strongly suggest that HspB8 efficiently contributes to target mutant ARpolyQ to autophagy, thus restoring a functional autophagic flux.

To strengthen our hypothesis, we also silenced LC3 with a specific shRNA. ShRNA effects were estimated using real-time PCR analysis showing that LC3 levels were reduced to almost a fourth (Fig. 6; approximately 75% reduction in the LC3 expression levels). LC3 silencing completely abolished the HspB8 prodegradative effects on ARpolyQ (AR.Q46) thus proving that HspB8 requires a functional autophagy to exert its protective antiaggregation and/or prodegradative functions.

Finally, to evaluate whether HspB8 exerts this “facilitator activity” on autophagy at very earlier stages (i.e., before misfolded protein insertion into autophagosomes), we analyzed mRNA level and protein distribution of p62. This protein acts in the very early stages of autophagic flux, because it recognizes ubiquitinated misfolded substrates, releasing them to autophagosomes.

As already mentioned, like LC3, endogenous p62 protein levels are also transcriptionally controlled and influenced by the rate of p62 protein clearance via autophagy. We then measured exogenous p62 levels (mCherry-p62), which are not transcriptionally influenced, but only affected by turnover (Klionsky et al., 2012). As shown in Fig. 6D, we found a considerable reduction of exogenous p62 when ARpolyQ was activated by testosterone. Proteasome inhibition has no major effect on p62 accumulation, and autophagy inhibition increased p62 accumulation. In contrast, HspB8 coexpression with ARpolyQ reduced exogenous p62 levels, in the absence and in the presence of testosterone.

Expression analysis (Fig. 6E) revealed that endogenous p62 mRNA levels increased in NSC34 cells overexpressing nonactivated ARpolyQ (AR.Q46) and HspB8, but decreased in the presence of testosterone-activated ARpolyQ and HspB8, in line with the data on endogenous LC3 mRNA expression and the permissive role of HspB8 on autophagy.

In the case of p62, when autophagic flux is insufficient (or impaired), p62 relocalizes accumulating into p62 bodies. We thus also analyzed the endogenous p62 protein distribution in the presence of ARpolyQ with or without testosterone (Fig. 6F). p62 distribution was affected by ARpolyQ independently of testosterone. In addition, p62 bodies did not colocalize with (or sequester) the nonactivated (soluble) ARpolyQ, and, a tight interaction between p62 bodies and testosterone-induced ARpolyQ aggregates was present. Of note, the two aggregated proteins remained physically independent (no cosegregation), suggesting that the recognition process of misfolded ARpolyQ by p62 is correctly initiated and the autophagic process started (see data on LC3 distribution and lipidation). We thus might postulate that ARpolyQ aggregates and/or p62 bodies might derive from an incomplete autophagic flux impaired at initial stages, before ARpolyQ insertion into autophagosomes, because ARpolyQ inclusion did not colocalize with or sequester LC3-II. Interestingly, ARpolyQ aggregates and/or p62 bodies were not present in cells overexpressing HspB8 (see Fig. 5B), and ARpolyQ levels were very low.

To gain further information on the relevance of p62 bodies formation in ARpolyQ clearance, we manipulated ARpolyQ aggregation with two selective AR modulators, cyproterone acetate and casodex. As previously shown, cyproterone acetate induces ARpolyQ aggregates characterized by irregular shape, and casodex does not cause ARpolyQ aggregation (Rusmini et al., 2007). Interestingly, cyproterone acetate-induced ARpolyQ aggregates have a much stronger capability to confine p62 into bodies than the testosterone-induced ARpolyQ regular spherical shape inclusions (Fig. 6F). Instead, when ARpolyQ clearance was facilitated with casodex (Rusmini et al., 2007), p62 bodies were not detectable, further supporting a possible correlation between the kinetic of ARpolyQ aggregates formation and their limited clearance through the autophagic pathway (Fig. 6F).

Collectively, these data confirm our hypothesis that HspB8 prevents ARpolyQ aggregation possibly by restoring its degradation at earlier stages of the autophagic pathway (acting possibly via a better target recognition).

### 3.4 Comparison of the effect of HspB8 on trehalose-induced autophagic ARpolyQ clearance

Recent studies have provided convincing evidence that ARpolyQ removal via autophagy can be beneficial in SBMA (Montie and Merry, 2009; Montie et al., 2009, 2011). Trehalose is an autophagic activator used in these studies. Thus we wanted: (1) to compare the effects of trehalose and HspB8; (2) to test whether these two factors might act in a synergistic and/or additive manner; and (3) evaluate whether trehalose might facilitate the autophagic process by inducing HspB8 expression.

We initially confirmed that, in our NSC34 motoneuronal model of SBMA, trehalose counteracts the testosterone-induced formation of ARpolyQ (AR.Q46) insoluble species (Fig. 7A). In basal conditions, trehalose almost doubled the amount of LC3 mRNA (Fig. 7B), and HspB8 was ineffective on LC3 transcription. Thus, though trehalose activates autophagy, HspB8 ameliorates the autophagic process but does not activate autophagy. These data were then further confirmed by measuring endogenous LC3-I and LC3-II protein levels in basal condition (nontransfected NSC34 cells). Fig. 7C shows that trehalose increased the conversion of LC3-I to its lipidated, autophagosome anchored, LC3-II, and HspB8 was not effective in this pathway. In immunofluorescence analysis, trehalose, but not HspB8, induced a mild increase of the levels of endogenous LC3-II and p62 proteins.

Then, we evaluated the trehalose effect on autophagy in the presence of ARpolyQ and we found that in the absence or in the presence of testosterone, trehalose increased mRNA levels of LC3 and p62 (Fig. 7D and E, respectively). Thus, the presence of a mutant misfolded protein sensitizes cells to a greater autophagic activation after trehalose treatment.

Finally, by blocking the autophagic pathway at very early stages with 3-MA, we confirmed that trehalose removes misfolded ARpolyQ insoluble species via autophagy (Fig. 7F). In fact, we found that 3-MA attenuates the effects of trehalose on the mutant AR, because a large accumulation of insoluble ARpolyQ can be detected in FRA in the presence of trehalose and 3-MA, and very low levels were detectable in the presence of trehalose alone.

**Fig. 8:**
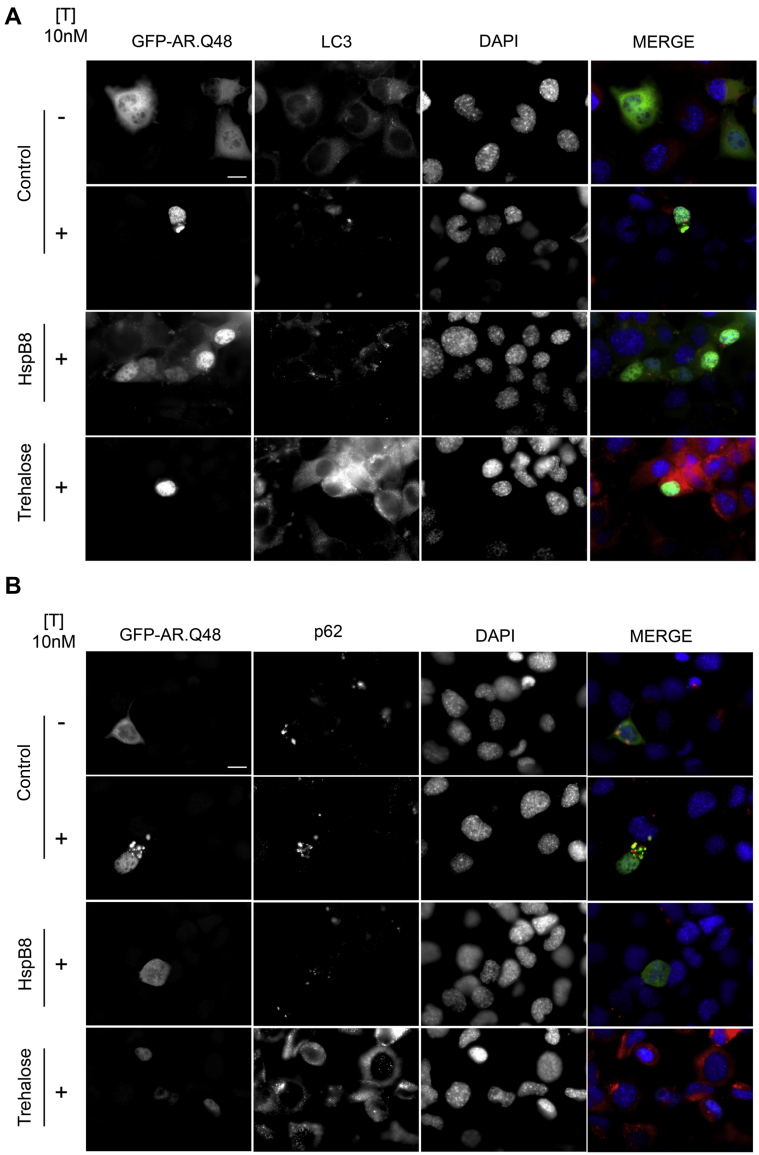
Heat shock protein (Hsp)B8 and trehalose exert different activities on the autophagic removal of androgen receptor (AR) gene with an abnormally long polyglutamine tract (ARpolyQ). (A) and (B) High resolution immunofluorescence analysis of NSC34 cells transfected with GFP-AR.Q48, pCDNA3, or HspB8 in the absence (−T) or in the presence (+T) of 10 nM of T for 48 hours, in basal condition or after treatment with 100 mM of trehalose for 48 hours. Nuclei were stained with DAPI (blue). Images obtained at magnification ×63 (scale bar, 10 μm). Abbreviations: DAPI, 4,6-diamidino-2-phenylindole; pCDNA3, empty vector; T, testosterone.

The data obtained by measuring LC3 mRNA and p62 mRNA expression and protein levels were paralleled by their protein distribution (Fig. 8A and B, respectively). In fact, both autophagic markers, LC3-II and p62 proteins, were found in a typical autophagy-associated distribution (punctate LC3-II distribution and disappearance of the ARpolyQ-induced p62 bodies). In agreement with the molecular (real-time PCR) and biochemical data (WB), trehalose induced a robust increase of LC3-II levels, further confirming that trehalose activates autophagy in immortalized motoneuronal cells (Fig. 8A).

**Fig. 9:**
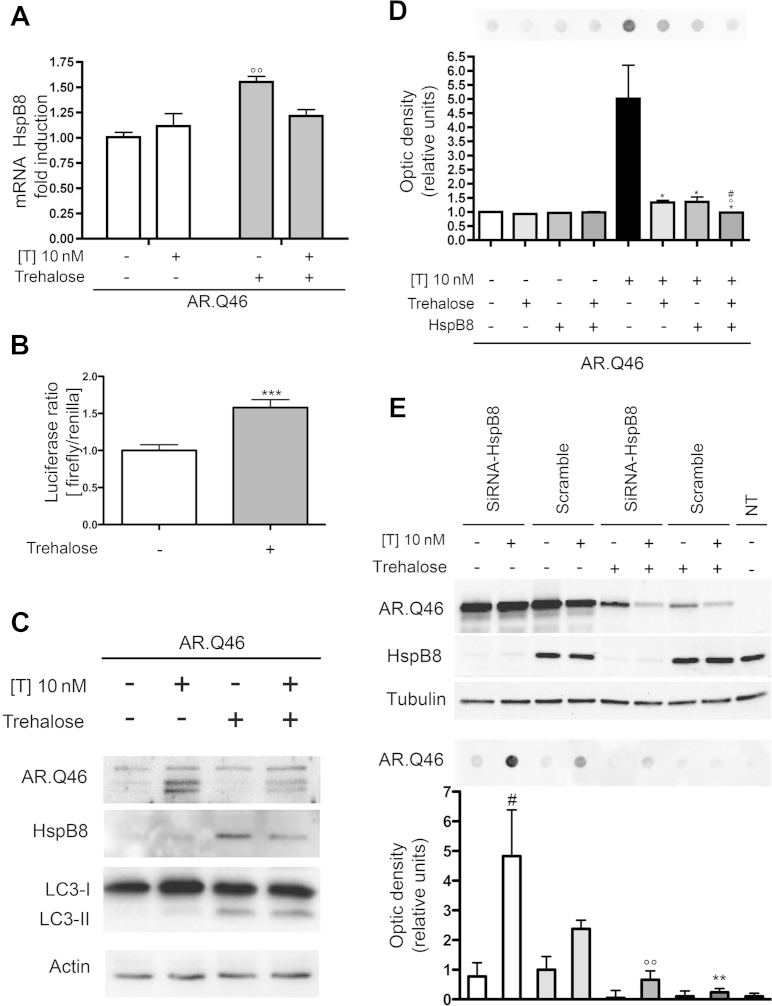
Trehalose induces heat shock protein (Hsp)B8 expression and both act in the autophagic clearance of mutant androgen receptor (AR) gene with an abnormally long polyglutamine tract (ARpolyQ). (A) Real-time polymerase chain reaction of HspB8 mRNA expression levels on NSC34 cells expressing AR.Q46 in the absence (−T) or in the presence (+T) of 10 nM of T for 48 hours, in basal condition or after treatment with 100 mM of trehalose for 48 hours (°° *p* < 0.01 vs. AR.Q46−T). (B) Transcriptional activity assay of HspB8 promoter performed on NSC34 cells transfected with promB8 and pRL-TK, in basal condition or after treatment with 100 mM of trehalose for 48 hours (*** *p* < 0.001 vs. untreated control). (C) Western blot analysis on cell lysates of NSC34 cells expressing AR.Q46 in the absence (−T) or in the presence (+T) of 10 nM of T for 48 hours, in basal condition or after treatment with 100 mM of trehalose for 48 hours. β-actin was used to normalize protein loading. (D) Filter retardation assay performed on NSC34 cells transfected with AR.Q46, pCDNA3, or HspB8, in the absence (−T) or in the presence (+T) of 10 nM of T for 48 hours, in basal condition or after treatment with 100 mM of trehalose for 48 hours (* *p* < 0.05 vs. AR.Q46+T; ° *p* < 0.05 vs. AR.Q46+T+trehalose; # *p* < 0.05 vs. AR.Q46+T+HspB8). (E) Upper insets, Western blot analysis, and lower insets, filter retardation assay, on cell lysates of NSC34 cotransfected with AR.Q46 and a small interfering RNA for endogenous HspB8 (SiRNA-HspB8) or its negative control (scramble). Cells were analyzed in the absence (−T) or in the presence (+T) of 10 nM of T for 48 hours, in basal condition or after treatment with 100 mM of trehalose for 48 hours. α-Tubulin was used to normalize protein loading (# *p* < 0.05 vs. scramble+T, °° *p* < 0.01 vs. scramble+T; °° *p* < 0.01 vs. SiRNA-HspB8+T). Abbreviations: mRNA, messenger RNA; NT, untransfected cells; pCDNA3, empty vector; pRL-TK, renilla luciferase expression vector; promB8, firefly luciferase controlled by HspB8 promoter; SiRNA, small interfering RNA; T, testosterone.

We found also that trehalose increased HspB8 mRNA (Fig. 9A) level. Moreover, taking advantage of the promB8 construct (Crippa et al., 2010a, 2010b), in which HspB8 promoter drives luciferase transcription, we demonstrated that trehalose effects on HspB8 are exerted at transcriptional levels (Fig. 9B). This also resulted in increased HspB8 protein level (Fig. 9C), suggesting that the autophagic response to trehalose might also be potentiated by a facilitation of autophagy, mediated by HspB8. We thus evaluated whether trehalose and HspB8 might act synergistically on the removal of misfolded ARpolyQ, measuring total insoluble ARpolyQ in FRA. The data (Fig. 9D) demonstrate that trehalose and HspB8 possess similar antiaggregation power on ARpolyQ, and their effects on testosterone-induced insoluble ARpolyQ species are increased when the 2 act together.

To evaluate whether HspB8 is involved in the pathways activated by trehalose to generate an increased autophagic response, we silenced HspB8, and measured the amount of ARpolyQ-insoluble species still present after trehalose treatment. The data shown in Fig. 9E confirmed the importance of HspB8 in the removal of testosterone-induced ARpolyQ-insoluble species (see also Fig. 1E). HspB8 silencing in the presence of trehalose resulted in a significant increase of the accumulation of ARpolyQ (AR.Q46)-insoluble species compared with trehalose alone. However, HspB8 silencing was not sufficient to completely abolish the prodegradative effects of trehalose on ARpolyQ-insoluble species, suggesting that HspB8 is a mediator, but not the only one, of the proautophagic activity of trehalose.

## 4 Discussion

In the present study we investigated the effect of HspB8 on the autophagic removal of the mutant ARpolyQ responsible for motoneuronal cell death in SBMA. In our immortalized motoneuronal cells, used as an SBMA model, we confirmed that ARpolyQ aggregates only after testosterone treatment and specifically in cell cytoplasm, as seen in some affected neuronal populations of SBMA patients (Adachi et al., 2005; Suzuki et al., 2008). While, ARpolyQ aggregation also occurs in the nuclei of cells from SBMA patients (Katsuno et al., 2010; Walcott and Merry, 2002), we could not detect nuclear inclusions in our SBMA model (Rusmini et al., 2011). In fact, reconstituting neuronal SBMA cell models with nuclear aggregates require a very long polyQ tract (>100 contiguous Qs) (Montie et al., 2009; Walcott and Merry, 2002). Indeed, ARpolyQ exerts its neurotoxicity mainly at the nuclear level (even if independently from inclusion formation), and cytoplasmic retention of ARpolyQ has been found to prevent most of its adverse effects on cell survival (Montie et al., 2009, 2011).

In our cells the levels of aberrant and/or potentially neurotoxic misfolded species of ARpolyQ (induced by testosterone) cannot be directly quantified. However, we took advantage of the fact that misfolded species accumulate into “testosterone-induced aggregates” and adopted aggregates as a “biomarker of ARpolyQ misfolding.”

Our initial approach was to evaluate whether HspB8 alters misfolded ARpolyQ dynamics. We found that HspB8 prevents the formation of testosterone-induced ARpolyQ cytoplasmic aggregates and facilitates ARpolyQ clearance from the cells. This clearance of ARpolyQ does not require a fully functional proteasome system. Silencing experiments have shown that the basal levels of HspB8 present in our cells are sufficient to stimulate the removal of most, but not all the misfolded ARpolyQ-insoluble species generated after testosterone treatment. Interestingly, we also found that, in the presence of ARpolyQ (either in absence or presence of testosterone; thus, nontoxic vs. toxic conformation, respectively), the autophagic process is already activated, because p62 and LC3 levels are increased with LC3-II in its punctate, autophagosome-linked, distribution. Thus, cytoplasmic aggregates accumulation is indicative of an incompleted autophagy, which can be associated with autophagic flux blockage. HspB8 facilitates the autophagy-mediated degradation of the mutant ARpolyQ, possibly by enhancing its recognition. Notably, when autophagy is pharmacologically or genetically blocked, HspB8 cannot assist ARpolyQ degradation. Thus, by preventing misfolded ARpolyQ accumulation, HspB8 might indirectly restore the autophagic flux to physiological level. HspB8 alone does not induce LC3 mRNA (and p62 mRNA, not shown) expression, and is unable to activate LC3 conversion to its lipidated, autophagosome-anchored LC3-II, 2 steps required for autophagy activation. Therefore, in our motoneuronal cells, HspB8 does not trigger autophagy, but it ameliorates the already activated autophagic process. In fact, others and we recently demonstrated that, in motoneuronal cells, HspB8 not only associated with its natural cochaperone BAG-3 (Carra et al., 2008a, 2008b), but also interacts (when bound to BAG-3) with Hsc70 and CHIP. This heteromeric complex is required to allow the CHIP-mediated ubiquination of the target misfolded substrates (Crippa et al., 2010b). CHIP dysfunction has also been reported in SBMA (and in amyotrophic lateral sclerosis [ALS]) and its overexpression has a beneficial effect in both diseases (Morishima et al., 2008). Similar observations have been reported for the other members of the hetero-complex, Hsc70 (Urushitani et al., 2004; Wang et al., 2009), or Hsp70 (Adachi et al., 2003; Kobayashi et al., 2000; Warrick et al., 1999; Waza et al., 2005, 2006). The same multiheteromeric complex has been found in muscle (Arndt et al., 2010), where it serves for the recognition of proteins damaged (misfolded) during prolonged physical exercise. It is of note that ARpolyQ might exert toxicity also at the muscle level (Adachi et al., 2007; Arndt et al., 2010; Cardozo et al., 2003; Jana et al., 2005; Johansen et al., 2009; Miller et al., 2005; Mo et al., 2010; Urushitani et al., 2004; Yu et al., 2011). When the substrate is ubiquitinated by CHIP, within the HspB8/BAG-3/Hsc70/CHIP complex, the misfolded protein can be released and recognized by p62 for its insertion into autophagosomes (Carra et al., 2012). The importance of cytoplasmic recognition and autophagic clearance of ARpolyQ to prevent its nuclear neurotoxicity has already been clearly established by other investigators (Montie and Merry, 2009; Montie et al., 2009, 2011). Based on this molecular model of the HspB8 mechanism of action, the mutant ARpolyQ has to be recognized in its misfolded fraction, after its release from accessory proteins (HSP70, HSP90, etc.) during the activation process (triggered by testosterone) (Poletti, 2004; Rusmini et al., 2007). Thus, once recognized by HspB8, the misfolded ARpolyQ will be rerouted from aggregation (and possibly from proteasomal degradation) to the “ubiquitination complex” of BAG-3, Hsc70, and CHIP, recognized by p62 and then released to the autophagic pathway. We found a close association of ARpolyQ aggregates with the p62 bodies, but not p62 sequestration; we did not find ARpolyQ aggregates that colocalized with (or were surrounded and/or entrapped by) LC3-II. Instead, LC3–II-positive/ARpolyQ-negative autophagosomes accumulate in cells. Collectively, these data indicate that autophagic flux alterations occur at very early stages, possibly during cytoplasmic misfolded protein recognition, but before the ARpolyQ insertion into the autophagosomes. In fact, during basal autophagy activation, p62 is mainly diffused and shows an elevated turnover. Instead, with defective autophagy (i.e., linked to impairment in cargo delivery or autophagic flux blockage), p62 polymerizes, accumulates, and aggregates into Triton X–100-insoluble p62 bodies (Mizushima and Komatsu, 2011). Notably, these bodies enhances alternative p62 functions to act as a scaffold protein in several signaling pathways (Matsumoto et al., 2011; Mizushima and Komatsu, 2011), that are crucial for the cell response to protein toxicity and also known to be able to trigger deleterious pathways for neuronal cells (Matsumoto et al., 2011; Mizushima and Komatsu, 2011). The data presented here clearly demonstrate that the overexpression of HspB8, either by facilitating the ARpolyQ recognition, or its ubiquitination by CHIP (or even the acquisition of a particular conformation more suitable for the interaction with the ubiquitination complex), prevents the p62 bodies formation (and thus their potential neurotoxicity) and the LC3-II accumulation in large (potentially deleterious) excess, allowing the complete removal of misfolded ARpolyQ via an autophagic flux restored to its physiological level. In addition, this “cytoplasmic” activity of HspB8 will prevent the migration of misfolded ARpolyQ species into the nuclei, where they can exert their neurotoxicity. So far, there is no evidence that HspB8 can exert effects in the nucleus, even if the protein is also localized in this intracellular compartment.

In a parallel study, we compared the effect of HspB8 on ARpolyQs with different polyQ sizes (Q46 and Q112), which aggregate in different cellular compartments in SBMA cell models. Indeed though AR.Q46 shows cytoplasmic aggregation, AR.Q112 shows nuclear aggregation. In that study we found that HspB8 is much less active in the removal of insoluble species generated by AR.Q112 than by AR.Q46 (Carra et al., 2013). Though aggregate localization (nuclear vs. cytoplasmic) might be involved in this selective sensitivity of HspB8 against the 2 ARpolyQs, it is also possible that particular AR.Q112 aggregate structures (or their faster kinetic of formation) make them less sensitive to the prodegradative activity of HspB8. Unfortunately, this observation might have a relevant implication in the attempt to confirm the beneficial effects of HspB8 in SBMA animal models. In fact, all transgenic (tg) SBMA mice showing neurological signs and presently available, bear a very long CAG stretch, coding for polyQ tracts of at least 97 Qs (Adachi et al., 2003; Chevalier-Larsen et al., 2004; Katsuno et al., 2003a, 2003b; La Spada et al., 1998; Li et al., 2007; Lieberman and Robins, 2008; Yu et al., 2006). This tract has never been found in patients, and might be more similar to that usually found in the huntingtin mutant protein associated with juvenile forms of Huntington disease, which are clinically distinct (and possibly have a different molecular basis of toxicity) from the forms found in older patients associated with shorter, but still pathological tracts (polyQs ranging from 36 to 60) (Martindale et al., 1998).

Previous studies performed in our lab, already showed that in spinal cord motoneurons that survive at the end stage of disease in transgenic ALS mice (Crippa et al., 2010a, 2010b), HspB8 is expressed at very high levels. Similar data have been obtained in spinal cord of ALS patients (Anagnostou et al., 2010). Finally, preliminary results obtained in our laboratory suggest that HspB8 expression is also increased at later stages of disease in the spinal cord of transgenic SBMA mice (knock-in mice developed by Prof Andrew Lieberman) (Jordan and Lieberman, 2008; Yu et al., 2006) (Rusmini et al, unpublished observation). Therefore, on the basis of the HspB8 mechanism of action proposed here, it is possible that HspB8 plays a protective role in affected motoneurons by facilitating the clearance of potentially neurotoxic misfolded proteins. It would be of great interest to find pharmacological approaches to overinduce HspB8 expression in motoneurons of affected individuals before appearance of symptoms. By analyzing the activation of the HspB8 promoter we found that it is positively regulated by proteasome inhibition (data confirmed also by WB analysis), a condition paralleled by a compensatory autophagy activation and triggered by misfolded proteins causative of neurodegenerative diseases (Crippa et al., 2010a, 2010b). This evidence correlates with the desaturation of UPS observed when HspB8 is expressed in the presence of nonactivated ARpolyQ (AR.Q46 without testosterone). However, as mentioned earlier in text, HspB8 does not directly trigger autophagy, therefore other rerouting mechanisms and/or proteins should be involved in the specific degradative pathways selected for the intracellular clearance of misfolded proteins (i.e., BAG-3). It must also be noted that the prodegradative and/or antiaggregation activity of HspB8 is also detectable using other protein prone to misfold and linked to different neurodegenerative diseases (Carra et al., 2005; Crippa et al., 2010a, 2010b).

Interestingly, our data are in line with very recent observations obtained in tg mice models of SBMA. In fact, the alteration of the overall chaperone response, obtained by knocking down HSF-1 expression in tg SBMA mice resulted in increased symptomatology and the involvement of structures normally unaffected in SBMA (Kondo et al., 2013). In contrast, overexpression of the Hsp70 interacting protein, which increases the Hsp70 substrate interaction, induces ARpolyQ clearance (Wang et al., 2013). Interestingly, it has recently been reported that a synthetic cochaperone mimicking Hsp70 interacting protein activity alleviates ARpolyQ toxicity in a SBMA *Drosophila* model (Wang et al., 2013). Moreover, upregulation or stimulation of the chaperone response, and of the mediators of autophagy involved in HspB8 activity (i.e., CHIP) ameliorates the symptomatology in the same tg SBMA mice models (Adachi et al., 2003, 2007; Carra et al., 2013; Katsuno et al., 2005; Kobayashi and Sobue, 2001; Kobayashi et al., 2000; Tokui et al., 2009; Waza et al., 2005, 2006). Finally, arimoclomol, a coinducer of the heat shock stress response, administered orally to tg SBMA mice significantly improved hind limb muscle force and contractile characteristics, rescuing motor units. Arimoclomol treatment also resulted in improved motor neuron survival, with increased expression of VEGF, an important neurotrophic factor. Thus is well accepted that heat shock response activation might have relevant therapeutic implications in SBMA (Malik et al., 2013).

We also analyzed the activity of the autophagy inducer, trehalose, which has been shown to be beneficial in SBMA (Montie and Merry, 2009; Montie et al., 2009, 2011) and other types of neurodegenerative diseases (Lan et al., 2012). Trehalose induced the LC3 expression and promoted the LC3-II formation in our immortalized motoneuronal cells, confirming that it activates autophagy. Interestingly, we found that trehalose increased the endogenous levels of HspB8 in motoneuronal cells activating the HspB8 promoter. These data suggest that the proautophagic activity of trehalose on ARpolyQ might also require HspB8 activation, even if silencing experiments have shown that HspB8 is a mediator, but not the only one involved in the proautophagic activity of trehalose. Hovewer, the two factors act synergistically in the removal of ARpolyQ-insoluble species induced by testosterone, indicating that potential therapeutic approaches to counteract ARpolyQ neurotoxicity in SBMA must taken into account in the possibility of using cocktails of drugs that might interfere with different steps of the pathogenic mechanism.
